# A Brief Analysis of Proteomic Profile Changes during Zebrafish Regeneration

**DOI:** 10.3390/biom12010035

**Published:** 2021-12-27

**Authors:** Zulvikar Syambani Ulhaq, William Ka Fai Tse

**Affiliations:** 1 Department of Biochemistry, Faculty of Medicine and Health Sciences, Maulana Malik Ibrahim State Islamic University of Malang, Batu 65144, Indonesia; zulhaq@kedokteran.uin-malang.ac.id; 2 National Research and Innovation Agency, Central Jakarta 10340, Indonesia; 3 Laboratory of Developmental Disorders and Toxicology, Center for Promotion of International Education and Research, Faculty of Agriculture, Kyushu University, Fukuoka 819-0395, Japan

**Keywords:** proteomic, regeneration, caudal fin, heart, reparative neurogenesis, zebrafish

## Abstract

Unlike mammals, zebrafish are capable to regenerate many of their organs, however, the response of tissue damage varies across tissues. Understanding the molecular mechanism behind the robust regenerative capacity in a model organism may help to identify and develop novel treatment strategies for mammals (including humans). Hence, we systematically analyzed the current literature on the proteome profile collected from different regenerated zebrafish tissues. Our analyses underlining that several proteins and protein families responsible as a component of cytoskeleton and structure, protein synthesis and degradation, cell cycle control, and energy metabolism were frequently identified. Moreover, target proteins responsible for the initiation of the regeneration process, such as inflammation and immune response were less frequently detected. This highlights the limitation of previous proteomic analysis and suggested a more sensitive modern proteomics analysis is needed to unfold the mechanism. This brief report provides a list of target proteins with predicted functions that could be useful for further biological studies.

## 1. Introduction

Regeneration is a process by which an organism restores or replaces damaged tissues through a complex mechanism, resulting in the same morphological and physiological properties as the undamaged one [1,2]. In mammals, several tissues such as skin and the gut epithelia exhibit a highly cellular turnover. On the other hand, neurons and cardiomyocytes are not completely restored [2,3]. Thus, limited regenerative capacity observed in mammals is believed to contribute to a variety of diseases, such as neurodegenerative disorders and heart failure. Additionally, regeneration in mammalian cells typically leads to scarring [4]. This possibly due to the imbalance between regeneration and scar formation [5,6].

In contrast to mammals, zebrafish exhibits a highly regenerative capacity in response to cellular damage [7]. Hence, it has been extensively emerged as a promising model for the study of regeneration [2,7,8,9]. Zebrafish regeneration studies are typically focused on adult tissues that include the spinal cord, brain, retina, caudal fin, and heart [2,8]. Although it is notable that stem/progenitor cells are crucial elements in eliciting regenerative response [4], other factors such as inflammatory mediators and extracellular matrix (ECM) play an important role during cellular remodeling [1,10]. Growing evidence shows that the mechanisms of regeneration are frequently examined by gene expression through several approaches, including PCR, *in situ* hybridization (ISH), microarray, and sequencing [11]. However, such results did not reflect protein levels. To fill such gap, we here analyze the available literature evaluating proteomic changes during zebrafish regeneration. This study provides hints to unlock the molecular mechanism of regenerative processes, which may be useful to explore a possible therapeutic approach as well as to expand our knowledge on regenerative medicine.

## 2. Methods

### 2.1. Literature Search

A literature search was conducted in PubMed and Scopus databases using the following search terms: “proteomic” (all fields), “regeneration” (all fields), and “zebrafish” (all fields), dated up to 31 December 2020. Titles and abstracts were screened for dataset inclusion. The final dataset consisted of 8 studies [1,3,11,12,13,14,15,16], which were further evaluated.

### 2.2. Group Definition

The studies were stratified according to the type of regenerating organs (including, central nervous system (brain, retina), caudal fin, and heart). Additional analysis was also performed by comparing different studies within a group if they consist of at least two studies.

### 2.3. Data Extraction and Analysis

Differentially expressed proteins reported in each study were included in the joint dataset with the following criteria: (1) Proteins were only counted once for each study with multiple spot identifications or evaluated in a different time series; (2) proteins were included if the expression level fold changes were below 0.8 or higher than 1.2. A Venn diagram was performed using InteractiVenn [17] to visualize the similarities and differences of the significant protein profiles among groups.

Additionally, to understand more detailed information into the role of the identified proteins required during zebrafish regeneration, we also looked at the biological processes and functions of all differentially expressed proteins in each experimental study.

## 3. Results and Discussion

### 3.1. Dataset Description

Proteomic alterations of the included studies were evaluated by 2-DiGE (Two-dimensional difference gel electrophoresis, *n* = 3) [12,14,15], LC-MS/MS (liquid chromatography-mass spectrometry, *n* = 3) [1,11,16], and SILAC (stable isotope labelling with amino acids in cell culture, *n* = 2) [3,13]. Proteomic analysis was examined from several regenerating organs, including the brain, retina, caudal fin, and heart. The complete lists of differentially expressed proteins extracted with the applied database information from the included studies are depicted in Supplementary Table S1.

**Figure 1:**
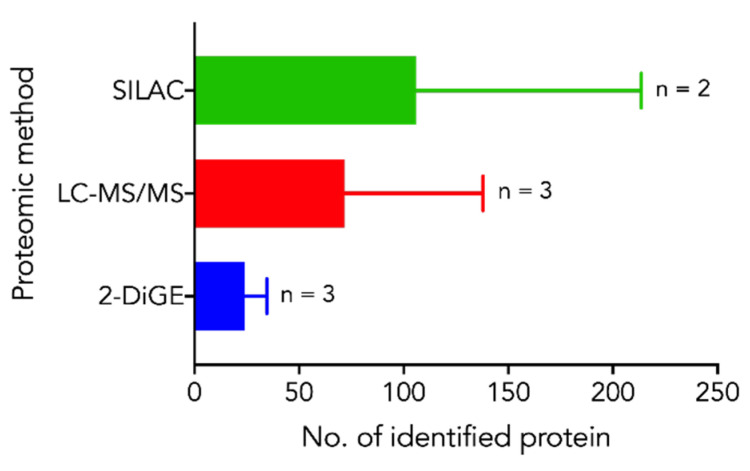
Differentially expressed proteins per study stratified by proteomic methods.

Of the analyzed studies, on average 62 differentially expressed proteins were identified. Although we did not find any significant number of protein profile changes between proteomic methods, 2-DiGE methods tended to have a lower number of significantly identified proteins (Figure 1).

### 3.2. Individual Proteins and Protein Families Repeatedly Regulated during Zebrafish Regeneration

**Figure 2:**
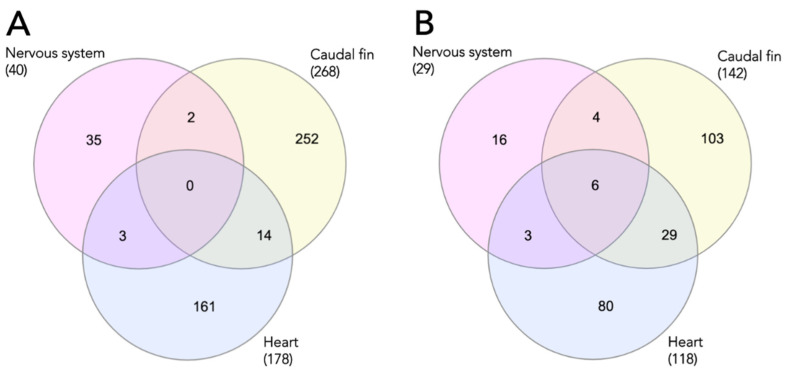
Venn diagram of the identified proteins (**A**) and its families (**B**) according to the regenerating organs.

Based on our grouping criteria, 486 unique proteins were included for further analysis (Figure 2A). By combining the outcome, none of the proteins was commonly shared among the three groups (Figure 2A). On the other hand, collagen (type I, alpha 1) and spectrin alpha (non-erythrocytic 1) were frequently identified (38%) among the repeatedly 19 identified proteins in the list (Table 1). Additionally, several other collagen proteins, such as type I (alpha 2) and type VI (alpha 3 and 4a), were also listed, followed by keratins (5, 8, and 18). These findings are in line with a previously reported study in mammals [18], indicating that structural components are often deregulated, regardless of the experimental types. If we consider the tissue types of these top 19 identified proteins, 17 of them were found in the heart (except the vimentin and keratin 5); followed by the caudal fin (16 proteins). Fibulin-1, galectin, and fibrinogen alpha chain were found in the heart but not in the caudal fin (Table 1).

We also further analyzed the frequently identified protein families among 8 studies, and we found that 6 families (actins, cytoskeletal keratins, glutathione transferases, ribosomal proteins, histones, and annexins) were detected in all three groups (Figure 2B, Table 2). Among the top 42 lists of frequently identified protein families, cytoskeletal keratins and annexins were listed in the two top positions (Table 2), which was in agreement with the top lists of human studies [18], implying a high similarity between mammals and fish. Regarding the types of the tissues, caudal fin replaced the heart as the most frequently identified tissue in terms of protein family. Specifically, fibulins and tubulins were the two families that could not be identified in the fin. On the other hand, lipid binding proteins (FABP type), vimentins, tubulins, and transferrins were not found in the heart (Table 2).

**Figure 3:**
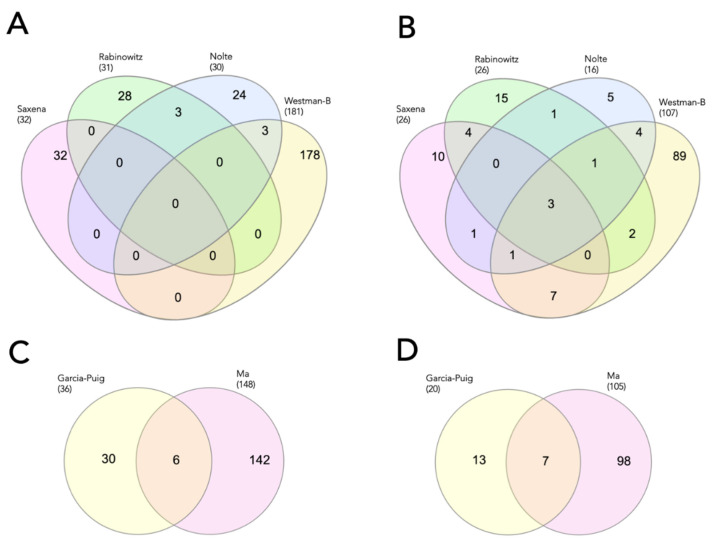
Venn diagram of the identified proteins and its families detected during caudal fin (**A**,**B**) and heart (**C**,**D**) regeneration, respectively. Studies are indicated by the name of the first author.

The four and two studies evaluating proteomic profile changes in regenerated caudal fin and heart were examined, yielding 274 and 184 unique proteins (Figure 3A,C) and 175 and 125 protein families (Figure 3B,D), respectively. None of the identified proteins observed in a group of the regenerated caudal fin was identical (Figure 3A) [3,13,14,16]. On the other hand, 6 proteins (fibrinogen (beta and gamma chains), collagen (type I and XII), fibronectin 1a, and cardiac myosin light chain-1) were detected in both studies of the regenerated heart [1,11]. Three (annexins, zgc, and hypothetical proteins) and seven (collagens, cytoskeletal keratins, actins, myosins, fibrinogens, fibronectins, and complement components) protein families were identified in all studies of the regenerated caudal fin and heart, respectively. Neither individual protein nor its family was identically detected in regenerated brain and retina (data not shown).

Utilizing the ISH technique, Martorana et al. successfully demonstrated that keratinocyte migration is a key factor for caudal fin regeneration [19]. Similarly, interkinetic nuclear migration modulated by the interaction between actins and myosins is required to replace photoreceptors damage [20]. Moreover, inhibition of myosin II disrupts the subcellular localization of crypto (epidermal growth factor-CFC) in facilitating cell-mediated wound healing in injured-caudal fin [21], thereby implying the interplay between myosin and cripto is crucial for stem cell proliferation.

Annexins functions are not limited to the membrane and structural complexes, but also influence zebrafish’s regenerating capacity [22]. Upregulation of *anxa2a* and *2b* transcripts and their proteins have been reported in regenerating caudal fin [22,23]. Additionally, the regulatory region of *anxa2a* and *2b* genes demonstrated the ability to repress histone methylations [23], and hence implicates transcriptional activation. This epigenetic regulatory mechanism may be necessary to drive a large number of genes that are required for various cellular events during the initiation of regeneration. Indeed, knocking down of *anxa2a* and *2b* hampered the regenerating capacity of zebrafish fin [22].

Another factor such as collagen seems to be involved in accelerating zebrafish regeneration [24]. Transient accumulation of collagen I and XII in the lesion site has been proven to contribute to the reparative matrixes production as well as a promoter for axonal or heart regeneration [25,26]. It is interesting to note that the localization of collagen XII is restricted in a few places of mammalian tissues [26,27]. On the other hand, its expression is highly distributed throughout the whole tissues of zebrafish [27]. Together, these results thus strengthen the notion that cytoskeletal proteins may be essential in modulating the higher regenerative capability observed in zebrafish.

### 3.3. Biological Processes and Functions among Identified Proteins

**Figure 4:**
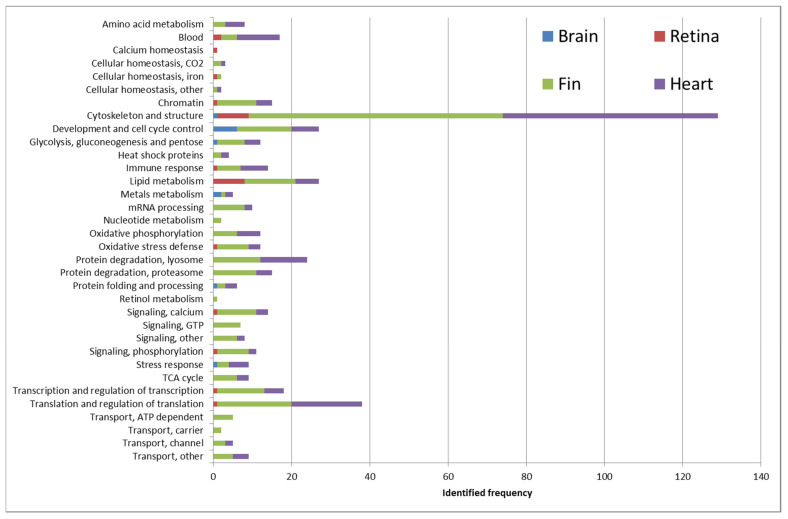
Proportions of biological processes and functions among differentially expressed proteins extracted from all included studies. The percentage was calculated from total proteins (499 proteins) reported in all studies.

We observed a total of 499 unique proteins across all analyzed studies, of which the top identified terms were cytoskeleton and structure, followed by protein synthesis and degradation, cell cycle control, and energy metabolism (Figure 4). This result is in accordance to our finding that the structural proteins predominantly (31%) occupied the top 42 lists of protein families among the included studies (Table 2). The top two identified protein families were the cytoskeleton and structure; and the translation and regulation of translation. These families have been suggested to play critical roles in tissue regeneration [28]. Since injury will stimulate the extracellular matrix activity via cell–cell and cell–matrix adhesions, which could be induced by various growth factors [29,30]. Afterwards, the regeneration process will include the cell proliferation, and the release of various inflammatory factors and cytokines such as tumor necrosis factor-alpha (TNF- α) and interleukin cytokines for tissue repair [31,32]. It should be noted that other regulatory proteins that initiate the regeneration process such as inflammation and/or immune response were detected at low levels. This is possibly associated with the limitation of current proteomic technologies. Another plausible explanation is that the inflammatory mediators and immune cells maybe transiently upregulated and then downregulated after regeneration process was initiated.

Moreover, the regulation of cytoskeleton and metabolic signaling pathways were involved in tissue regeneration for the cell movement, growth, and proliferation [33,34]. Studies showed that the modification of lipid metabolism is needed in liver and axon regeneration [35,36]. Lastly, the assembly and activity of ribosomal proteins are required for protein synthesis and involved in regeneration process [37,38]. The identification of such protein families as the top enriched terms among the zebrafish regeneration studies confirmed the importance of these mechanisms during regeneration.

Regarding the mammalian regeneration proteomics studies, a review paper from deer concluded the differentially expressed proteins were mainly responsible for multiple biological processes and signaling pathways. Among them, cytoskeleton was highly spotted in various studies [39]. Another review paper summarized the mammalian liver regeneration further suggesting terms such as the cell–cell contact, and cytokines were highly identified [28]. Regarding the cardiac progenitor cells and pluripotent stem cell derived cardiomyocytes, similar enriched terms such as metabolism, cytoskeleton, and cell adhesion could be spotted [40]. On the other hand, numerous terms were specifically identified in the various models, which were expected as the regeneration ability between the zebrafish and mammal are different. Nevertheless, using the zebrafish as the regeneration model has the advantages of identifying target molecules to provide therapeutic strategies for repairing the mammalian tissues.

Despite being the first systematic analysis in the field, several limitations of this study should be noted. First, the outcome of different proteomic techniques varied. This might be the results of biological and methodological differences (e.g., type of tissue/organ, time of evaluation, sample preparation, and type of data analysis). Second, only a limited number of studies were included for further evaluation. Consequently, more studies are still warranted to test our findings with a larger dataset. Third, global proteomic analysis is more likely to miss a target biomarker for specific regenerated tissues. Forth, the identified proteins might be induced by the injury but not responsible for the regeneration process.

Moreover, in terms of the proteomics technologies, there are several limitations in identifying the low-abundant proteins, such as several kinds of cytokines and growth factors. For example, the DIGE method is a powerful tool in evaluating proteomic profiles. However, the number of pitfalls should be taken into consideration, for example, poor visualization of low-copy number proteins and difficulty to separate protein with very large (>150 kDa) and very small (<10 kDa) protein size [41]. Similarly, LC-MS/MS is also less sensitive for detection of small peptides e.g., cytokines [42]. Although SILAC is a suitable technique for quantitative proteomics, its applicability for cytokine measurement is limited because it cannot be used to directly label tissues or body fluids [43]. Hence, antibody-based techniques should be performed simultaneously to facilitate a systematic examination of the proteomic studies in regenerated zebrafish. These limitations could be improved by modified extraction tools and methods [44,45]. Together with the technological advancement on LC-MS and bioinformatics software, it is easier to identify qualified proteins in these days. Regarding the quantification, the Isobaric Tag for Relative and Absolute Quantitation (iTRAQ) has been widely used in mammalian studies [46]. On the other hand, the application in fishes is still very limited. Several studies have used the iTRAQ in fish physiology and environmental studies [47,48]; however, it has not been widely used in the regeneration studies. Furthermore, another isobaric multiplex, Tandem Mass Tag (TMT) could further extend the number of testing samples to 16 [49]. Lastly, we noticed an increasing usage of the data independent acquisition (DIA) mass spectrometry in biological research. This method is claimed to cover most of the low abundance and small peptides [50]. DIA identifies peptides within the selected m/z windows, and thus has merits like low missing value and good data reproducibility [51,52]. In addition, the protein identification can be performed by various searching engines, such as DIA-Umpire PECAN [53], or DirectDIA [51], but not limited to the conventional genome-wide species-specific database that is used in traditional MS/MS ion mass spectrum. All these advantages suggest that the DIA will become another popular proteomics tool in the near future. To conclude, the current advancement of the proteomics could be a powerful tool for identifying the proteins participating in regeneration process.

## 4. Conclusions

This study demonstrated that the structural proteins are commonly detected or deregulated with functions like cytoskeleton organization, protein synthesis and degradation, cell cycle control, and energy metabolism during zebrafish regeneration. On the other hand, target proteins responsible for the initiation of the regeneration process, such as inflammation and immune response were less frequently detected. Further functional research should be performed to find specific targets that are responsible for modulating the regenerative capacity in fish. To summarize, the studies provide a set of gene list that is potentially useful to enhance the regeneration process.

## Figures and Tables

**Table 1:** List of the top 19 proteins repeatedly identified in the selected studies. F as caudal fin, H as heart; R as retina; B as brain.

Rank Position	Protein Name	Among the 8 Selected Studies	
		Total	Organs
1–2	Collagen, type I, alpha 1	3	F, H
1–2	Spectrin alpha, non-erythrocytic 1	3	F, H
3–19	Vimentin	2	R, F
3–19	Coactosin-like 1	2	F, H
3–19	Nucleoside diphosphate kinase	2	F, H
3–19	Cathepsin B	2	F, H
3–19	Fibulin-1	2	B, H
3–19	Galectin	2	R, H
3–19	Fibrinogen alpha chain	2	R, H
3–19	Keratin 5	2	R, F
3–19	Collagen, type I, alpha 2	2	F, H
3–19	Collagen, type VI, alpha 3	2	F, H
3–19	Collagen, type VI, alpha 4a	2	F, H
3–19	Periostin, osteoblast specific factor	2	F, H
3–19	Keratin, type II cytoskeletal 8	2	F, H
3–19	Keratin, type I cytoskeletal 18	2	F, H
3–19	Caveolae-associated protein 1b	2	F, H
3–19	Lamin A	2	F, H
3–19	60S acidic ribosomal protein P2	2	F, H

**Table 2:** List of the top 42 protein families repeatedly identified in the selected studies. F as caudal fin, H as heart; R as retina; B as brain.

Rank Position	Protein Family	Among the 8 Selected Studies	
		Total	Organs
1–2	Cytoskeletal keratins	6	R, F, H
1–2	Annexins	6	R, F, H
3–5	Actins	5	R, F, H
3–5	Hypothetical proteins	5	F, H
3–5	Zgc	5	F, H
6–12	Glutathione transferases	4	R, F, H
6–12	Ribosomal proteins	4	R, F, H
6–12	Protein phosphatases	4	F, H
6–12	Myosins	4	F, H
6–12	Histones	4	R, F, H
6–12	Lipid binding proteins (FABP type)	4	R, F
6–12	Collagens	4	F, H
13–20	Elongation factors	3	F, H
13–20	Heat shock proteins	3	F, H
13–20	Carbonic anhydrases	3	F, H
13–20	Fibrinogens	3	R, H
13–20	Lamins	3	F, H
13–20	Spectrins	3	F, H
13–20	Complement components	3	F, H
13–20	Peptidases	3	F, H
21–42	Coactosins	2	F, H
21–42	Peroxiredoxins	2	F, H
21–42	Nucleoside diphosphate kinases	2	F, H
21–42	Cofilins	2	F, H
21–42	Cathepsins	2	F, H
21–42	Fibulins	2	B, H
21–42	Galectins	2	R, H
21–42	Vimentins	2	R, F
21–42	Tubulins	2	R, F
21–42	Transferrins	2	R, F
21–42	Periostins	2	F, H
21–42	Cavins	2	F, H
21–42	Adenylyl cyclase-associated proteins	2	F, H
21–42	ATP synthase subunits	2	F, H
21–42	Adaptor complexes medium subunits	2	F, H
21–42	COX subunits	2	F, H
21–42	HD lipoprotein-binding proteins	2	F, H
21–42	Integrins	2	F, H
21–42	NADH dehydrogenases	2	F, H
21–42	Plakophilins	2	F, H
21–42	Sex hormone-binding globulins	2	F, H
21–42	Thioredoxins	2	F, H

## Data Availability

All data generated or analyzed during this study are included in this published article (and its supplementary information files).

## Supplementary Materials

The following supporting information can be downloaded at: https://www.mdpi.com/article/10.3390/biom12010035/s1, Table S1: Extracted data and characteristics of the included studies.
